# Differences in the frequency of subjective geriatric complaints along with aging and their associations with physical function, multimorbidity, and mood: A cross-sectional study

**DOI:** 10.1371/journal.pone.0263889

**Published:** 2022-02-11

**Authors:** Hajime Takechi, Akira Tsuzuki, Komaki Matsumoto, Akane Fukui, Hitomi Kawakita, Hiroshi Yoshino, Yoshikiyo Kanada

**Affiliations:** 1 Department of Geriatrics and Cognitive Disorders, School of Medicine, Fujita Health University, Toyoake, Aichi, Japan; 2 Faculty of Rehabilitation, School of Health Science, Fujita Health University, Toyoake, Aichi, Japan; 3 Department of Community Care, Toyoake City Municipal Office, Toyoake, Aichi, Japan; 4 Faculty of Human Health Science, Graduate School of Medicine, Kyoto University, Kyoto, Japan; Ehime University Graduate School of Medicine, JAPAN

## Abstract

**Background:**

In this study, we investigated subjective geriatric complaints (SGCs) as conditions regarding health concerns in community-dwelling older people and analyzed their frequencies with aging and relationships with other factors.

**Methods:**

This cross-sectional study enrolled 10,434 older people living in a community with a representative aging population in Japan. A questionnaire was sent by mail to those who had not applied for formal care needs certification. The presence of and concern for symptoms common in old age were asked as SGCs, as were physical function levels, multimorbidity, and depression. Categorical principal component analysis (CATPCA) of the symptoms was performed, and the association between the obtained components and other factors was analyzed.

**Results:**

The mean age of the participants was 73.7 ± 6.1 years, and 52.5% were women. On average, they had 1.72 ± 1.57 SGCs, which showed a gradual increase with age. The results of the CATPCA revealed four components: SGC1, excretory/circulatory/swallowing complaints; SGC2, audiovisual complaints; SGC3, neurological complaints; and SGC4, musculoskeletal complaints. All SGC components were independently associated with physical function, multimorbidity, and depression.

**Conclusions:**

Each SGC showed various frequencies and differences along with aging, and SGCs were classified into four components that were thought to share a common background. These findings could contribute to the planning of better health management strategies for older people.

## Introduction

The aging of the population and advances in medical treatment have resulted in an increasing number of older people with concurrent multiple diseases and conditions. The co-occurrence of multiple chronic or acute diseases in an individual is known as multimorbidity, and this has recently become an important issue [1]. In addition, some conditions are difficult to define as a single disease because of multiple factors associated with age-related changes in various organs and systems; these conditions are called geriatric syndromes and include symptoms such as urinary incontinence, pressure sores, delirium, and falls [2–5], which are sometimes referred to as geriatric giants because they have been a major challenge in terms of quality of life (QOL) among older people [6].

The concept of geriatric syndromes is very important in geriatric medicine, and their presence has been shown to be a prognostic factor in emergency hospitalization among the aged [7–9]. However, a wide variety of symptoms and conditions occurring in the context of aging have been called geriatric syndromes, and the symptoms and conditions involved differ depending on several aspects [8, 10, 11], ranging from symptoms that occur in older people who are living independently without nursing care to conditions such as delirium and pressure ulcers that occur in poor health conditions.

Recently, frailty has been noted as an intermittent condition between robustness and disability and a risk factor for geriatric syndromes [12–16]. It is recognized to be an important condition in old age. There are two main definitions of frailty, one using a phenotype with five indices and the other using the percentage of accumulation of deficits in physical and mental function and activities of daily living [12, 13, 17, 18]. In some studies, the symptoms associated with geriatric syndromes and frailty have been combined into geriatric conditions, and increased vulnerabilities to impairments in activities of daily living and a poor prognosis after emergency hospitalization with geriatric conditions have been observed [19, 20]. Therefore, multimorbidity, geriatric syndromes, frailty, and geriatric conditions can be viewed as a series of changes that converge along several different paths in the aging process [14, 21–23].

Although the health consequences associated with aging mentioned above are widely known, many older adults continue to live independently in their community while being concerned about the various symptoms associated with aging. These symptoms, designated here as subjective geriatric complaints (SGCs), consist of common age-related symptoms that community-dwelling older people experience in daily life. While some of these may overlap with those of geriatric syndromes, e.g., vision impairment and urinary incontinence, we assume that SGCs can be distinguished from geriatric syndromes from their clinical characteristics. For example, these symptoms remain relatively stable and would not have a major influence on the independence of older people. It has also been suggested that the aged may perceive health problems at a prodromal stage [24, 25]. The SGCs presented here could represent a critical concept capable of reflecting the vulnerability of older people at an earlier stage. Therefore, to contribute to the planning of better health management strategies for older people, this study aimed to identify the characteristics of SGCs and investigate their relationships with other age-related factors.

## Methods

### Participants

As part of the TOyoake Integrated Care Study (TOICS), a questionnaire was sent to 14,850 people aged 65 years or older living in Toyoake city (89.2% of the older people in the city) in a suburb of metropolitan Nagoya city, Japan [26]. No participant had applied for long-term care certification [27]. Responses were received from 10,740 people (response rate: 72.4%). Older community-dwelling people who had long-term care certification and were thought to be more vulnerable were excluded from the study (n = 1801, 10.8% of older people in the city). This survey was conducted in December 2016. After collecting the questionnaires via post, we removed the personal numbers that identified the individuals and created a data set for the analysis. After removing respondents with missing data for the main items, 10,434 people were finally analyzed. This study was approved by the bioethics review committee of the Fujita Health University School of Medicine (HM17-245). Written informed consent was obtained from all participants.

### Questionnaire

The questionnaire was composed of items on age, sex, family composition, body mass index, physical function, multimorbidity, and depressive mood. With regard to SGCs, in the questionnaire, we asked, “Which of the following symptoms may interfere with your daily life?” and listed 14 symptoms: dizziness, headache, abdominal pain, insomnia, urination disorder (pollakiuria and/or urinary incontinence), defecation disorder (constipation and/or diarrhea), vision impairment, hearing loss, appetite loss, low back pain, arthralgia (including tingling), dysphagia (including choking), shortness of breath, and edema. These lists of symptoms were constructed according to existing lists of geriatric syndromes and related publications [2, 8, 28–30]. Symptoms and conditions classified as being part of a discrete disease, such as dementia, were excluded from the list. Those often accompanied by severe illness not observed in community-dwelling older people living independently, such as delirium or pressure ulcers, were also excluded from the list. After a preliminary analysis, we excluded abdominal pain from further analysis because it accounted for less than 5% of the participants in any age group. Multiple responses for SGCs were allowed. The number of SGCs in each person was designated as the SGC score (0–13).

Regarding physical function, we asked the following three questions related to physical ability from the Kihon Checklist (KCL) [31]: (1) “Do you normally climb stairs without using a handrail or wall for support”, (2) “Do you normally stand up from a chair without any aids?”, and (3) “Do you normally walk continuously for 15 minutes?”. The number of “yes” answers for each person was designated as the physical function score (0–3). Regarding multimorbidity, the numbers of the following diseases were counted: hypertension, stroke, heart disease, diabetes, hyperlipidemia, respiratory disease, gastrointestinal disease, renal/urinary tract disease, musculoskeletal disease, trauma, malignant tumor, hematologic and immune disease, ophthalmologic disease, and otolaryngology disease. The number of diseases was designated as the multimorbidity score (0–14). We evaluated depression using two questions on depressive mood and loss of interest in regular activities [32]. We considered a “yes” answer to either question as positive for depression.

### Statistical analyses

The participants’ characteristics, SGC score, physical function score, multimorbidity score, and depression status were summarized using basic descriptive statistics. The mean, standard deviation (SD), and proportion were used as appropriate. Differences in basic characteristics among age groups were compared using one-way analysis of variance for continuous variables and chi-squared tests for categorical variables. To show aging-related changes in 13 SGCs, the frequency (%) of symptoms was indicated based on six age groups (65–69, 70–74, 75–79, 80–84, 85–89, and 90 years and older). The difference in frequencies in younger age groups (65–69, 70–74, and 75–79 years) and older age groups (80–84, 85–89, and 90 years and older) was shown based on the ratio of frequencies in both groups.

Categorical principal components analysis (CATPCA) with varimax rotation was performed to examine the factor structure of the SGCs since the responses to the questionnaire were nominal. To select the optimal number of components, the Kaiser criterion (based on eigenvalue ≥1) was employed. Items with factor loading >.30 were retained. Multiple regression analysis was conducted to evaluate the association between each SGC component and physical function, multimorbidity, and depressive mood. All statistical analyses were carried out using IBM SPSS for Windows (ver. 27.0; IBM, Armonk, NY). A two-tailed *P* value of < .05 was considered significant.

## Results

The mean age of the participants was 73.7 ± 6.1 years, and 52.5% were women. The physical function scores for each age group (in 5-year increments) decreased with age (2.46 ± 0.84 in the 65–69 to 1.43 ± 1.10 in the 90 years and older group). On the other hand, the multimorbidity score increased with age (1.16 ± 1.13 in the 65–69 to 1.90 ± 1.36 in the 90 years and older group), as did the SGC score (1.47 ± 1.39 in the 65–69 to 2.26 ± 1.78 in the 90 years and older group) and proportion of higher numbers of SGCs. On average, 21% of the participants did not have subjective complaints. Rates of depression did not change across age groups (Table 1).

**Table 1:** Characteristics of the study participants.

			Age group (years)												
	Total (n = 10,434)		65–69 (n = 3286)		70–74 (n = 2887)		75–79 (n = 2374)		80–84 (n = 1312)		85–89 (n = 462)		>90 (n = 113)		
	Mean	SD	Mean	SD	Mean	SD	Mean	SD	Mean	SD	Mean	SD	Mean	SD	*P*
Age (years)	73.7	6.1	67.2	1.4	72.2	1.4	76.8	1.4	81.7	1.4	86.4	1.3	91.6	1.9	< .001
Women (%)	52.5		53.8		53.3		50.1		51.7		51.3		60.2		.03
BMI	22.6	3.1	22.7	3.2	22.7	3.1	22.5	3.2	22.4	3.0	22.1	3.1	21.6	3.8	< .001
Physical function score	2.29	0.95	2.46	0.84	2.39	0.90	2.24	0.96	1.99	1.05	1.74	1.12	1.43	1.10	< .001
Depression (%)	34.0		33.4		32.6		34.3		37.1		36.1		37.2		.07
Multimorbidity score	1.45	1.27	1.16	1.13	1.41	1.24	1.63	1.31	1.75	1.34	1.82	1.44	1.90	1.36	< .001
SGC score	1.72	1.57	1.47	1.39	1.61	1.54	1.84	1.57	2.13	1.77	2.29	1.89	2.26	1.78	< .001
Number of SGCs (%)															
0	21.0		25.3		23.2		17.8		14.0		13.9		15.9		< .001
1	33.3		35.3		34.0		32.9		29.7		28.4		24.8		
2	22.1		21.5		21.9		22.9		24.5		17.7		21.2		
3	11.4		9.6		10.5		12.4		13.4		18.6		13.3		
4	6.2		4.4		5.0		7.5		8.4		11.7		12.4		
≥5	6.0		3.9		5.4		6.5		10.0		9.7		12.4		

Differences among age groups were compared using one-way analysis of variance for continuous variables and chi-squared tests for categorical variables. BMI: body mass index, SGC: subjective geriatric complaint, SD: standard deviation.

**Fig 1:**
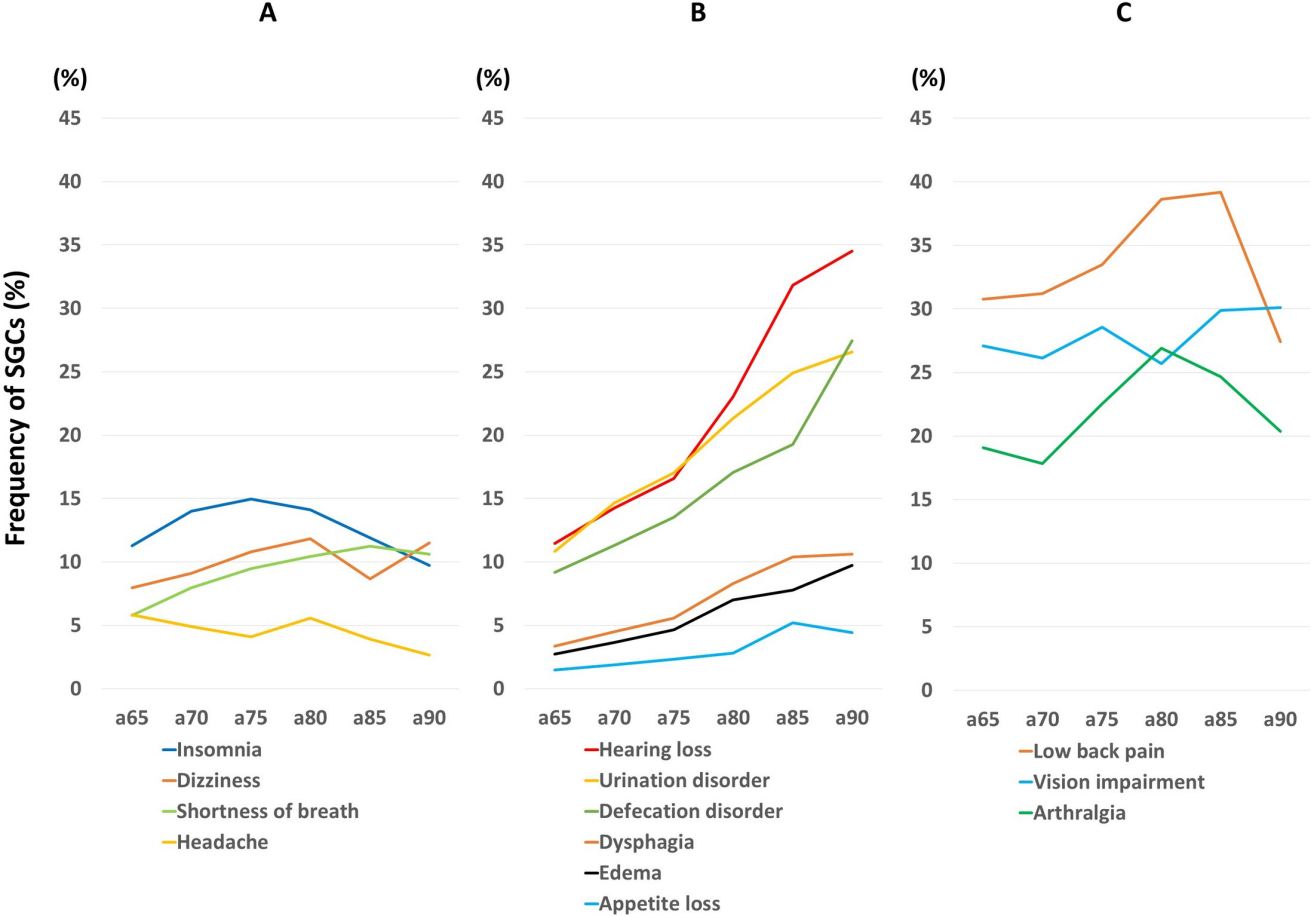
Frequency of subjective geriatric complaints (SGCs). (A) Four SGCs showed a relatively low frequency, with 15% or less than 15% in all age groups. (B) Three SGCs (appetite loss, dysphagia, and edema) showed a relatively low frequency, but steeply increased with age; the ratio of older/younger age groups was > 1.5. Three other SGCs (defecation disorder, urination disorder, and hearing impairment) showed a relatively high frequency, with more than 15% in all age groups, and steeply increased with age. (C) Three SGCs were frequent in all age groups, with rates of 15% or more in each. The horizontal axis indicates the age group. Each 5-year age group is shown with ‘a’ plus youngest age in the group, e.g., a65 means the age group of 65–69 years.

The frequencies of SGCs are indicated in Table 2 and Fig 1. Four SGCs (headache, shortness of breath, dizziness, and insomnia) showed a relatively low frequency, with 15% or less than 15% in all age groups (Fig 1A). Three other SGCs (appetite loss, dysphagia, and edema) also showed a relatively low frequency, but steeply increased with age; the ratio of older/younger age groups was > 1.5 (Fig 1B). Three SGCs (defecation disorder, urination disorder, and hearing impairment) showed a relatively high frequency, with more than 15% in all age groups, and steeply increased with age (Fig 1B). Three SGCs (arthralgia, vision impairment, and low back pain) were frequent in all age groups, with rates of 15% or more in all age groups (Fig 1C).

**Table 2:** Changes in the frequency of symptoms with aging.

	Age group (years)									
	65–69	70–74	75–79	80–84	85–89	≥90	Average of % in all age groups	Average of % in younger age groups (Y)	Average of % in older age groups (O)	Ratio O/Y
	n = 3286	n = 2887	n = 2374	n = 1312	n = 462	n = 113				
Headache	5.8	4.9	4.1	5.6	3.9	2.7	4.5	4.9	4.0	0.8
Shortness of breath	5.8	8.0	9.5	10.4	11.3	10.6	9.3	7.7	10.8	1.4
Dizziness	8.0	9.1	10.8	11.8	8.7	11.5	10.0	9.3	10.7	1.1
Insomnia	11.3	14.0	15.0	14.1	11.9	9.7	12.7	13.4	11.9	0.9
Appetite loss	1.5	1.9	2.3	2.8	5.2	4.4	3.0	1.9	4.1	2.2
Dysphagia	3.3	4.5	5.6	8.3	10.4	10.6	7.1	4.5	9.8	2.2
Edema	2.7	3.6	4.6	7.0	7.8	9.7	5.9	3.7	8.2	2.2
Defecation disorder	9.2	11.3	13.5	17.1	19.3	27.4	16.3	11.3	21.3	1.9
Urination disorder	10.8	14.7	17.0	21.3	24.9	26.5	19.2	14.2	24.3	1.7
Hearing loss	11.4	14.3	16.6	23.0	31.8	34.5	21.9	14.1	29.8	2.1
Arthralgia	19.1	17.8	22.5	26.9	24.7	20.4	21.9	19.8	24.0	1.2
Vision impairment	27.1	26.2	28.6	25.7	29.9	30.1	27.9	27.3	28.5	1.0
Low back pain	30.8	31.2	33.5	38.6	39.2	27.4	33.5	31.8	35.1	1.1
Average of % of all symptoms	11.3	12.4	14.1	16.4	17.6	17.4	14.9	12.6	17.1	1.5

Numbers in the table indicate the frequency (%) of each symptom in the designated age group and the ratio of the frequency (only in the rightmost column) in the older age group divided by the younger age group (Ratio O/Y). The younger age groups include the age groups of 65–69, 70–74, and 75–79 years. The older age groups include the age groups of 80–84, 85–89, and 90 years and over.

Then, CATPCA was performed to examine the factor structure of the SGCs. CATPCA based on the responses of 13 SGCs in 10,434 individuals aged 65 years and older revealed four components: SGC1, excretory/circulatory/swallowing complaints; SGC2, audiovisual complaints; SGC3, neurological complaints; and SGC4, musculoskeletal complaints (Table 3). SGC1 included six symptoms and SGC2, SGC3, and SGC4 included two symptoms.

**Table 3:** Categorical principal component analysis (CATPCA) yielded four components.

	SGC1	SGC2	SGC3	SGC4
Defecation disorder	**0.552**	–0.072	–0.067	0.202
Urination disorder	**0.53**	0.177	–0.31	0.107
Edema	**0.476**	–0.002	0.104	0.021
Shortness of breath	**0.459**	0.125	0.241	–0.197
Dysphagia	**0.399**	0.258	0.098	0.037
Appetite loss	**0.39**	–0.109	0.265	–0.066
Insomnia	0.291	0.033	0.269	0.151
Hearing loss	0.033	**0.762**	–0.016	0.016
Vision impairment	0.038	**0.737**	0.076	0.028
Headache	–0.007	0.022	**0.699**	0.166
Dizziness	0.151	0.081	**0.655**	–0.022
Low back pain	–0.014	0.035	0.087	**0.75**
Arthralgia	0.134	0.023	0.05	**0.682**
Eigenvalues	1.88	1.174	1.074	1.029
Variation (%)	14.46	9.033	8.264	7.914
Total variation (%)	39.671			

Factor loadings higher than 0.3 are shown in bold. SGC: subjective geriatric complaint.

Multiple regression analysis using the number of SGCs in individuals in each of the four components as the dependent variable and physical function scores, multimorbidity scores, and depression as independent variables was performed to identify associations. The results revealed that all four SGC components were significantly independently associated with physical function scores, multimorbidity scores, and depression after adjusting for age and gender (Table 4).

**Table 4:** Association between each SGC component and other factors.

**SGC1 and related factors**			
	β	*t*	*P*
Physical function score	–0.141	–14.702	< .001
Multimorbidity score	0.22	23.187	< .001
Depression	0.128	13.734	< .001
**SGC2 and related factors**			
	β	*t*	*P*
Physical function score	–0.033	–3.289	.001
Multimorbidity score	0.13	12.941	< .001
Depression	0.092	9.328	< .001
**SGC3 and related factors**			
	β	*t*	*P*
Physical function score	–0.062	–6.162	< .001
Multimorbidity score	0.089	8.876	< .001
Depression	0.139	14.181	< .001
**SGC4 and related factors**			
	β	*t*	*P*
Physical function score	–0.16	–16.099	< .001
Multimorbidity score	0.153	15.534	< .001
Depression	0.09	9.276	< .001

SGC: subjective geriatric complaint.

## Discussion

In this study, we characterized the symptoms of health concerns as SGCs among older people living in the community who were not receiving long-term nursing care. Changes in the frequency of SGCs with aging revealed SGCs with a low frequency of complaints in all age groups, SGCs with a steep increase with age, and SGCs with a high frequency of complaints that did not show a noticeable change with age. CATPCA was performed to examine the relationships between SGCs and physical function, multimorbidity, and depressive mood, and the following four components were obtained: SGC1, excretory/circulatory/swallowing complaints; SGC2, audiovisual complaints; SGC3, neurological complaints; and SGC4, musculoskeletal complaints. Each component was associated with multimorbidity, physical function, and depressive mood.

Geriatric syndromes are well-known conditions that occur with aging. The classical geriatric syndromes are associated with the so-called “giants of old age” [2, 3, 5, 6, 11]. In recent decades, various efforts have been made in regard to geriatric syndromes to improve the QOL of older people, since these conditions severely degrade their QOL. However, the advancing aging of the population has led to a number of challenges for older people. The numbers of conditions and symptoms included in geriatric syndromes have been increasing accordingly and the boundary of geriatric syndromes has become obscure [8, 10, 11, 33, 34]. This study attempted to isolate SGCs as complaints that could be observed in relatively healthy older people living in the community who are not receiving long-term nursing care.

Multimorbidity and related polypharmacy may also be a major health challenge for the aged [1, 34–36]. It is conceivable that the SGCs of the aged may be associated with certain organ diseases. Older adults have been reported to be aware of health concerns before the onset of an apparent disease [24, 25, 37]. However, as the present CATPCA results revealed, older people are not necessarily concerned about their health in relation to a pathophysiologically associated disease, but are presumed to have complaints related to excretory/circulatory/swallowing, sensory, neurological, and musculoskeletal systems associated with physical function, multimorbidity, and depressive mood, suggesting that SGCs are not related simply to reasonably explainable organ-specific diseases. In addition, although the four patterns obtained in the present CATPCA are similar in some respects to those obtained in the analysis of multimorbidity, there are also clear differences between the patterns. In this regard, the relationship between SGCs and multimorbidity needs to be investigated further [38, 39].

In recent years, the concept of frailty has been proposed for the early detection of conditions leading to the need for nursing care in old age, and preventive interventions have been undertaken [12, 13, 22, 23, 31, 40]. The concept of frailty involves two major models: the phenotype model and the accumulation of deficits model [12, 13, 17, 18]. In the accumulation of deficits model, the conditions, symptoms, and functional decline that occur in old age are counted to evaluate frailty [13, 17, 18]. The SGCs presented in this study are assumed to be complaints at an earlier stage compared with the elements of frailty. Similar to the subjective cognitive decline proposed in relation to the early stages of dementia and the fear of falls proposed in relation to falls [37, 41], SGCs are intended to capture subjective perceptions of potential threats to health, rather than the disease or objective condition itself. Such a subjective condition can be clarified by conducting a questionnaire on older adults living in the community, but it may not be medically recognized as a symptom, and it may not lead to consultation behavior. In addition, frailty is usually indicated as a comprehensive indicator, while SGCs may be important in terms of both individual factors and as a comprehensive concept, similar to a disease and multimorbidity. Although the overlap among SGCs, frailty, and multimorbidity needs to be investigated in more detail, it is important to consider SGCs as an independent concept.

**Fig 2:**
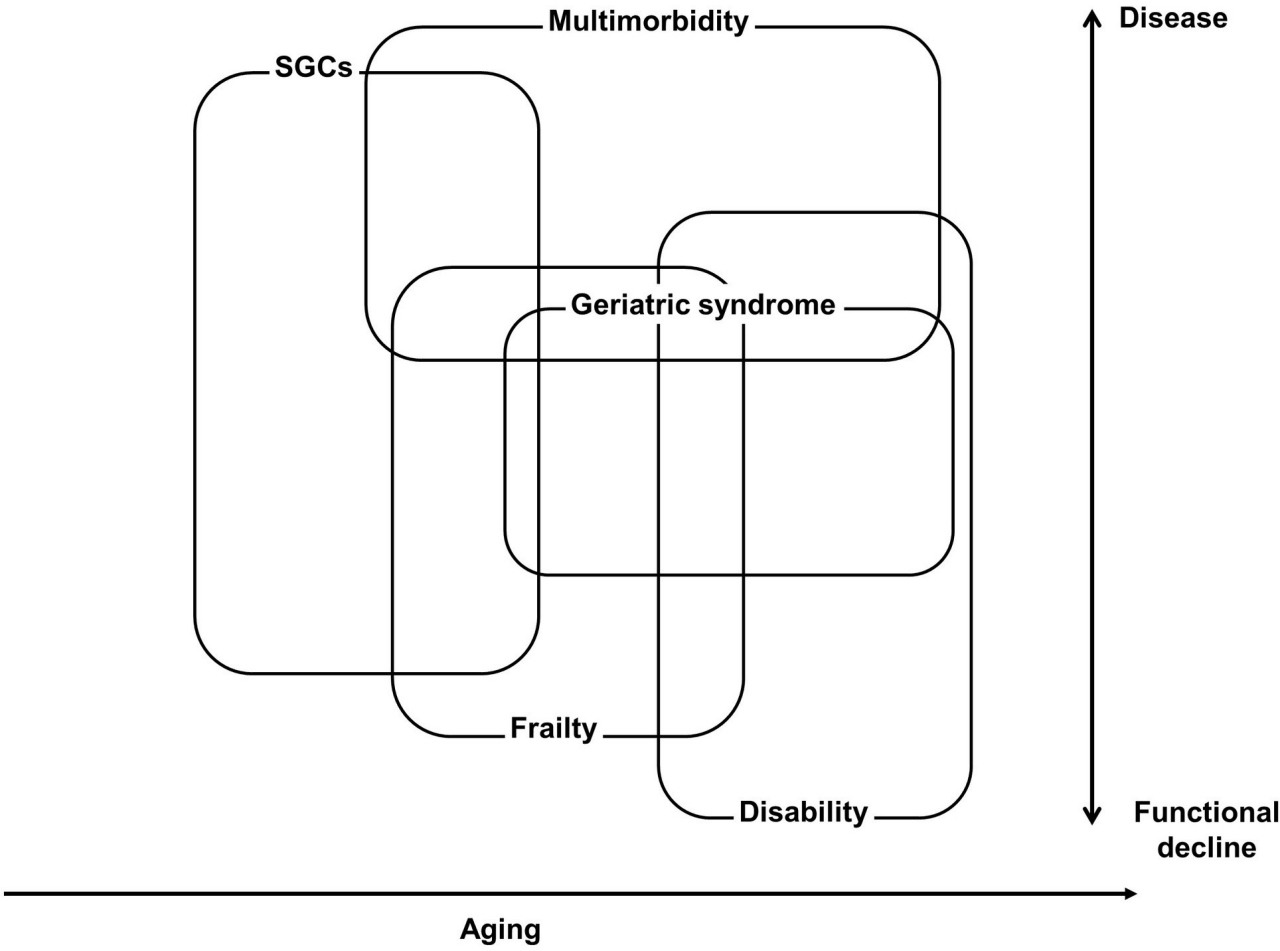
Hypothetical scheme of the relationships among SGCs, frailty, multimorbidity, geriatric syndrome, and disability. SGCs: subjective geriatric complaints.

Based on the results of the present study and related previous studies regarding geriatrics [2, 14, 21–23, 35, 40], we propose a hypothetical scheme for the relationships among SGCs, frailty, multimorbidity, geriatric syndrome, and disability, which is shown in Fig 2. It is also considered that, in this study, participants were community-dwelling older people who were almost independent in their daily lives and did not apply for long-term care certification. Community-dwelling older people who had care certification and were excluded from this study were older (81.6 ± 8.3 years compared to 73.7 ± 6.1 years of the participants in this study) and suspected to have more diseases and classical geriatric syndromes, be more frail and more disabled, because the care certification in Japan is determined after evaluating physical, mental or cognitive status and disability.

Although we used three items of the KCL as the physical function score in this study, it was assumed to be associated with frailty [31]. Depressive mood might be also involved in the scheme, as was shown in this study, but this was not included because its relationship with aging was not consistent. Several other studies have reported that symptoms in older people are related to poor life outcomes [42–45]. The multiple factors included in each category in the scheme may be understood as a cascade of interconnected changes with age. Some of these can be described as a process that can be interpreted as that of a single disease, whereas others can be classified as classical geriatric syndromes in which complex conditions manifest as a single phenomenon. Factors included in the scheme can be understood as a spectrum from a disease category that requires medical intervention to a disability category that requires nursing support. While most of the pathologies that occur in aging are considered to be progressive and irreversible, some factors may be considered preventable, reversible through rehabilitation and exercise, medically treatable, and amenable to non-pharmacological interventions, such as environmental adjustments and psychological care. Although all of the symptoms and conditions in this scheme could be included in geriatric syndromes in the broadest sense, since they might all be associated with aging, it is important to classify them to construct effective intervention strategies.

In Japan, the aging rate (age 65 years or over) has reached nearly 30%, and the average life expectancy for both men and women is over 80 years, making it an important issue to determine what health issues arise among the aged and when. As the global population is also aging, we believe that the situation in Japan is also important internationally. In this study, conducted jointly by medical professionals and policy makers, we were aware of the need to raise awareness and intervene at the right time to address such issues among the local population.

This study had several limitations. First, it was a cross-sectional survey, and we were unable to show an association between SGCs and longitudinal outcomes. In the future, it will be desirable to clarify the significance of SGCs by conducting a longitudinal analysis of the QOL associated with SGCs. Second, since this was a questionnaire-based survey, physical measurements and examinations were not performed. Future cross-sectional analyses and longitudinal studies of SGCs incorporating the actual measurement of some items would be important for a more detailed analysis. Third, we did not examine how the symptoms described as SGCs are related to actual diseases. For example, it would be desirable to investigate in detail the relationship between each SGC and individual diseases, such as whether complaints of visual impairment occur in presbyopia, cataracts, or age-related macular degeneration.

In conclusion, analyzing the complaints and health concerns of older people living in the community as SGCs may help to plan more effective interventions from an earlier stage and lead to the prevention of multimorbidity, frailty, and geriatric syndromes.

## References

[1] Barnett K, Mercer SW, Norbury M, Watt G, Wyke S, Guthrie B. Epidemiology of multimorbidity and implications for health care, research, and medical education: a cross-sectional study. Lancet. 2012;380(9836):37–43. Epub 2012/05/15. doi: 10.1016/S0140-6736(12)60240-2 . 22579043

[2] Inouye SK, Studenski S, Tinetti ME, Kuchel GA. Geriatric syndromes: clinical, research, and policy implications of a core geriatric concept. J Am Geriatr Soc. 2007;55(5):780–91. Epub 2007/05/12. doi: 10.1111/j.1532-5415.2007.01156.x ; PubMed Central PMCID: PMC2409147. 17493201 PMC2409147

[3] Tinetti ME, Inouye SK, Gill TM, Doucette JT. Shared risk factors for falls, incontinence, and functional dependence. Unifying the approach to geriatric syndromes. JAMA. 1995;273(17):1348–53. Epub 1995/05/03. . 7715059

[4] Lee PG, Cigolle C, Blaum C. The co-occurrence of chronic diseases and geriatric syndromes: the health and retirement study. J Am Geriatr Soc. 2009;57(3):511–6. Epub 2009/02/04. doi: 10.1111/j.1532-5415.2008.02150.x . 19187416

[5] Olde Rikkert MG. Conceptualizing geriatric syndromes. In: Michel J, Beattie B, Martin F, Walston J, editors. Oxford Textbook of Geriatric Medicine. 3rd ed: OUP Oxford; 2017. p. 355–62.

[6] Isaacs B. The Challenge of Geriatric Medicine: Oxford Medical Publications; 1992.

[7] Anpalahan M, Gibson SJ. Geriatric syndromes as predictors of adverse outcomes of hospitalization. Internal medicine journal. 2008;38(1):16–23. Epub 2007/06/05. doi: 10.1111/j.1445-5994.2007.01398.x . 17542997

[8] Flacker JM. What is a geriatric syndrome anyway? J Am Geriatr Soc. 2003;51(4):574–6. Epub 2003/03/27. doi: 10.1046/j.1532-5415.2003.51174.x . 12657087

[9] Lakhan P, Jones M, Wilson A, Courtney M, Hirdes J, Gray LC. A prospective cohort study of geriatric syndromes among older medical patients admitted to acute care hospitals. J Am Geriatr Soc. 2011;59(11):2001–8. Epub 2011/11/19. doi: 10.1111/j.1532-5415.2011.03663.x . 22092231

[10] Olde Rikkert MG, Rigaud AS, van Hoeyweghen RJ, de Graaf J. Geriatric syndromes: medical misnomer or progress in geriatrics? The Netherlands journal of medicine. 2003;61(3):83–7. Epub 2003/05/27. . 12765229

[11] Won CW, Yoo HJ, Yu SH, Kim CO, Dumlao LCI, Dewiasty E, et al. Lists of geriatric syndromes in the Asian-Pacific geriatric societies. Eur Geriatr Med. 2013;4(5):335–8. doi: 10.1016/j.eurger.2013.07.005

[12] Fried LP, Tangen CM, Walston J, Newman AB, Hirsch C, Gottdiener J, et al. Frailty in older adults: evidence for a phenotype. J Gerontol A Biol Sci Med Sci. 2001;56(3):M146–56. Epub 2001/03/17. doi: 10.1093/gerona/56.3.m146 . 11253156

[13] Rockwood K, Mitnitski A. Frailty in relation to the accumulation of deficits. J Gerontol A Biol Sci Med Sci. 2007;62(7):722–7. Epub 2007/07/20. doi: 10.1093/gerona/62.7.722 . 17634318

[14] Walston J, Buta B, Xue QL. Frailty Screening and Interventions: Considerations for Clinical Practice. Clin Geriatr Med. 2018;34(1):25–38. Epub 2017/11/14. doi: 10.1016/j.cger.2017.09.004 ; PubMed Central PMCID: PMC5726589. 29129215 PMC5726589

[15] Hubbard RE, Peel NM, Samanta M, Gray LC, Mitnitski A, Rockwood K. Frailty status at admission to hospital predicts multiple adverse outcomes. Age Ageing. 2017;46(5):801–6. Epub 2017/05/23. doi: 10.1093/ageing/afx081 . 28531254

[16] Syddall H, Roberts HC, Evandrou M, Cooper C, Bergman H, Aihie Sayer A. Prevalence and correlates of frailty among community-dwelling older men and women: findings from the Hertfordshire Cohort Study. Age Ageing. 2010;39(2):197–203. Epub 2009/12/17. doi: 10.1093/ageing/afp204 ; PubMed Central PMCID: PMC3546311. 20007127 PMC3546311

[17] Searle SD, Mitnitski A, Gahbauer EA, Gill TM, Rockwood K. A standard procedure for creating a frailty index. BMC Geriatr. 2008;8:24. Epub 2008/10/02. doi: 10.1186/1471-2318-8-24 ; PubMed Central PMCID: PMC2573877. 18826625 PMC2573877

[18] Theou O, O’Connell MD, King-Kallimanis BL, O’Halloran AM, Rockwood K, Kenny RA. Measuring frailty using self-report and test-based health measures. Age Ageing. 2015;44(3):471–7. Epub 2015/02/18. doi: 10.1093/ageing/afv010 ; PubMed Central PMCID: PMC4411224. 25687601 PMC4411224

[19] Cigolle CT, Langa KM, Kabeto MU, Tian Z, Blaum CS. Geriatric conditions and disability: the Health and Retirement Study. Ann Intern Med. 2007;147(3):156–64. Epub 2007/08/08. doi: 10.7326/0003-4819-147-3-200708070-00004 . 17679703

[20] Buurman BM, Hoogerduijn JG, de Haan RJ, Abu-Hanna A, Lagaay AM, Verhaar HJ, et al. Geriatric conditions in acutely hospitalized older patients: prevalence and one-year survival and functional decline. PLoS One. 2011;6(11):e26951. Epub 2011/11/24. doi: 10.1371/journal.pone.0026951 ; PubMed Central PMCID: PMC3215703. 22110598 PMC3215703

[21] Xue QL. The frailty syndrome: definition and natural history. Clin Geriatr Med. 2011;27(1):1–15. Epub 2010/11/26. doi: 10.1016/j.cger.2010.08.009 ; PubMed Central PMCID: PMC3028599. 21093718 PMC3028599

[22] Dent E, Martin FC, Bergman H, Woo J, Romero-Ortuno R, Walston JD. Management of frailty: opportunities, challenges, and future directions. Lancet. 2019;394(10206):1376–86. Epub 2019/10/15. doi: 10.1016/S0140-6736(19)31785-4 . 31609229

[23] Thillainadesan J, Scott IA, Le Couteur DG. Frailty, a multisystem ageing syndrome. Age Ageing. 2020;49(5):758–63. Epub 2020/06/17. doi: 10.1093/ageing/afaa112 . 32542377

[24] Beauchet O, Launay CP, Merjagnan C, Kabeshova A, Annweiler C. Quantified self and comprehensive geriatric assessment: older adults are able to evaluate their own health and functional status. PLoS One. 2014;9(6):e100636. Epub 2014/06/27. doi: 10.1371/journal.pone.0100636 ; PubMed Central PMCID: PMC4072604. 24968016 PMC4072604

[25] Hirosaki M, Okumiya K, Wada T, Ishine M, Sakamoto R, Ishimoto Y, et al. Self-rated health is associated with subsequent functional decline among older adults in Japan. Int Psychogeriatr. 2017;29(9):1475–83. Epub 2017/06/01. doi: 10.1017/S1041610217000692 . 28560936

[26] Takechi H, Tsuzuki A, Matsumoto K, Matsunaga S, Nishiyama H, Ogawa M, et al. Relationship between subjective memory complaints and social and leisure activities in community-dwelling older people: Toyoake Integrated Care Study. Geriatr Gerontol Int. 2020;20(10):867–72. Epub 2020/07/30. doi: 10.1111/ggi.13992 . 32725916

[27] Tsutsui T, Muramatsu N. Care-needs certification in the long-term care insurance system of Japan. J Am Geriatr Soc. 2005;53(3):522–7. Epub 2005/03/04. doi: JGS53175 [pii] doi: 10.1111/j.1532-5415.2005.53175.x . 15743300

[28] Hale WE, Perkins LL, May FE, Marks RG, Stewart RB. Symptom prevalence in the elderly. An evaluation of age, sex, disease, and medication use. J Am Geriatr Soc. 1986;34(5):333–40. Epub 1986/05/01. doi: 10.1111/j.1532-5415.1986.tb04315.x . 3958407

[29] Hoy D, Bain C, Williams G, March L, Brooks P, Blyth F, et al. A systematic review of the global prevalence of low back pain. Arthritis and rheumatism. 2012;64(6):2028–37. Epub 2012/01/11. doi: 10.1002/art.34347 . 22231424

[30] Jarrett PG, Rockwood K, Carver D, Stolee P, Cosway S. Illness Presentation in Elderly Patients. Arch Intern Med. 1995;155(10):1060–4. doi: 10.1001/archinte.1995.00430100086010 7748049

[31] Sewo Sampaio PY, Sampaio RA, Yamada M, Arai H. Systematic review of the Kihon Checklist: Is it a reliable assessment of frailty? Geriatr Gerontol Int. 2016;16(8):893–902. Epub 2016/07/23. doi: 10.1111/ggi.12833 . 27444395

[32] Whooley MA, Avins AL, Miranda J, Browner WS. Case-finding instruments for depression. Two questions are as good as many. J Gen Intern Med. 1997;12(7):439–45. Epub 1997/07/01. doi: 10.1046/j.1525-1497.1997.00076.x ; PubMed Central PMCID: PMC1497134. 9229283 PMC1497134

[33] Sanford AM, Morley JE, Berg-Weger M, Lundy J, Little MO, Leonard K, et al. High prevalence of geriatric syndromes in older adults. PLoS One. 2020;15(6):e0233857. Epub 2020/06/06. doi: 10.1371/journal.pone.0233857 ; PubMed Central PMCID: PMC7274399. 32502177 PMC7274399

[34] Stevenson JM, Davies JG, Martin FC. Medication-related harm: a geriatric syndrome. Age Ageing. 2019;49(1):7–11. Epub 2019/10/31. doi: 10.1093/ageing/afz121 . 31665207

[35] Kuzuya M. Era of geriatric medical challenges: Multimorbidity among older patients. Geriatr Gerontol Int. 2019;19(8):699–704. Epub 2019/08/10. doi: 10.1111/ggi.13742 . 31397060

[36] Aoki T, Yamamoto Y, Ikenoue T, Onishi Y, Fukuhara S. Multimorbidity patterns in relation to polypharmacy and dosage frequency: a nationwide, cross-sectional study in a Japanese population. Scientific reports. 2018;8(1):3806. Epub 2018/03/02. doi: 10.1038/s41598-018-21917-6 ; PubMed Central PMCID: PMC5830504. 29491441 PMC5830504

[37] Jonker C, Geerlings MI, Schmand B. Are memory complaints predictive for dementia? A review of clinical and population-based studies. Int J Geriatr Psychiatry. 2000;15(11):983–91. Epub 2000/12/13. doi: 10.1002/1099-1166(200011)15:11<983::aid-gps238>3.0.co;2-5 . 11113976

[38] Prados-Torres A, Calderón-Larrañaga A, Hancco-Saavedra J, Poblador-Plou B, van den Akker M. Multimorbidity patterns: a systematic review. J Clin Epidemiol. 2014;67(3):254–66. Epub 2014/01/30. doi: 10.1016/j.jclinepi.2013.09.021 . 24472295

[39] Violan C, Foguet-Boreu Q, Flores-Mateo G, Salisbury C, Blom J, Freitag M, et al. Prevalence, determinants and patterns of multimorbidity in primary care: a systematic review of observational studies. PLoS One. 2014;9(7):e102149. Epub 2014/07/23. doi: 10.1371/journal.pone.0102149 ; PubMed Central PMCID: PMC4105594. 25048354 PMC4105594

[40] Zamudio-Rodríguez A, Letenneur L, Féart C, Avila-Funes JA, Amieva H, Pérès K. The disability process: is there a place for frailty? Age Ageing. 2020;49(5):764–70. Epub 2020/05/05. doi: 10.1093/ageing/afaa031 . 32365166

[41] Murphy SL, Williams CS, Gill TM. Characteristics associated with fear of falling and activity restriction in community-living older persons. Journal of the American Geriatrics Society. 2002;50(3):516–20. doi: 10.1046/j.1532-5415.2002.50119.x 11943049 PMC3046411

[42] Patel KV, Guralnik JM, Phelan EA, Gell NM, Wallace RB, Sullivan MD, et al. Symptom Burden Among Community-Dwelling Older Adults in the United States. J Am Geriatr Soc. 2019;67(2):223–31. Epub 2018/12/15. doi: 10.1111/jgs.15673 ; PubMed Central PMCID: PMC6367017. 30548453 PMC6367017

[43] Salanitro AH, Hovater M, Hearld KR, Roth DL, Sawyer P, Locher JL, et al. Symptom burden predicts hospitalization independent of comorbidity in community-dwelling older adults. J Am Geriatr Soc. 2012;60(9):1632–7. Epub 2012/09/19. doi: 10.1111/j.1532-5415.2012.04121.x ; PubMed Central PMCID: PMC3458585. 22985139 PMC3458585

[44] Whitson HE, Sanders LL, Pieper CF, Morey MC, Oddone EZ, Gold DT, et al. Correlation between symptoms and function in older adults with comorbidity. J Am Geriatr Soc. 2009;57(4):676–82. Epub 2009/04/28. doi: 10.1111/j.1532-5415.2009.02178.x ; PubMed Central PMCID: PMC2674624. 19392960 PMC2674624

[45] Henchoz Y, Büla C, Guessous I, Rodondi N, Goy R, Demont M, et al. Chronic symptoms in a representative sample of community-dwelling older people: a cross-sectional study in Switzerland. BMJ Open. 2017;7(1):e014485. Epub 2017/01/18. doi: 10.1136/bmjopen-2016-014485 ; PubMed Central PMCID: PMC5253546. 28096256 PMC5253546

